# The Expression of Autophagy-Associated Genes Represents a Valid Footprint for Aggressive Pancreatic Neuroendocrine Neoplasms

**DOI:** 10.3390/ijms24043636

**Published:** 2023-02-11

**Authors:** Sami Matrood, Leander Edwin Melms, Detlef Klaus Bartsch, Pietro Di Fazio

**Affiliations:** 1 Department of Gastroenterology, Endocrinology, Metabolism and Infectiology, Philipps-University Marburg, 35043 Marburg, Germany; 2 Institute for Artificial Intelligence, University Hospital Marburg, Philipps-University Marburg, 35043 Marburg, Germany; 3 Department of Visceral, Thoracic and Vascular Surgery, Philipps-University Marburg, 35043 Marburg, Germany

**Keywords:** pancreatic neuroendocrine neoplasm, pancreatic neuroendocrine tumor, cancer, MEN1, autophagy, mTOR, beclin1

## Abstract

Pancreatic neuroendocrine neoplasms (pNEN) are rare and heterogeneous tumors. Previous investigations have shown that autophagy can be a target for cancer therapy. This study aimed to determine the association between the expression of autophagy-associated gene transcripts and clinical parameters in pNEN. In total, 54 pNEN specimens were obtained from our human biobank. The patient characteristics were retrieved from the medical record. RT-qPCR was performed to assess the expression of the autophagic transcripts *BECN1*, *MAP1LC3B*, *SQSTM1*, *UVRAG*, *TFEB*, *PRKAA1*, and *PRKAA2* in the pNEN specimens. A Mann–Whitney U test was used to detect differences in the expression of autophagic gene transcripts between different tumor characteristics. This study showed that G1 sporadic pNEN have a higher expression of autophagic genes compared to G2. Lymphatic and distant metastasis occurred significantly more often in pNEN with a decreased expression of the autophagic genes. Within sporadic pNEN, the insulinomas express higher levels of autophagic transcripts than gastrinomas and non-functional pNEN. MEN1-associated pNEN show a higher expression of autophagic genes than sporadic pNEN. In summary, a decreased expression of autophagic transcripts distinguishes metastatic from non-metastatic sporadic pNEN. The significance of autophagy as a molecular marker for prognosis and therapy decisions needs to be further investigated.

## 1. Introduction

Pancreatic neuroendocrine neoplasms (pNEN) arise from neuroendocrine cells of the pancreas and form a heterogeneous group of rare diseases with an increasing incidence in recent decades [1]. PNEN are classified into well-differentiated pancreatic neuroendocrine tumors (pNET) and poorly differentiated pancreatic neuroendocrine carcinomas (pNEC). PNET are classified by the Ki-67 index into G1, G2, and G3, according to the World Health Organization classification. PNEC are already G3 by definition [2]. PNEN occurs sporadically and can also develop in the context of endocrine syndromes, such as multiple endocrine neoplasia type 1 (MEN1) syndrome [3].

Autophagy is a physiological degradation process of the cell that serves to recycle unneeded and dysfunctional proteins and organelles through a lysosome-dependent process which was first described in the 1960s [4]. Initially, autophagy was identified as a mechanism that compensates the metabolic stress caused by nutrient deficiency [5]. At the end of the 1990s, the involvement of the autophagy process in malignancies has become evident [6]. Autophagy can block tumorigenesis by compensating for cellular stress, thus leading to cell death. On the other hand, autophagy can trigger cancer cell survival [7]. Additionally, autophagy can be used by tumor cells to develop resistance to therapeutics or to promote immune evasion [8,9,10].

Autophagy is still largely unexplored in pancreatic neuroendocrine neoplasms. The use of chloroquine as an autophagy inhibitor caused the overcoming of an autophagy-mediated resistance mechanism against sunitinib in the Rip1Tag2 mouse model [9]. However, the induction of autophagy by the pan-deacetylase inhibitor panobinostat led to autophagic cell death. Thus representing a therapeutic strategy for pNEN [11].

Various clinical parameters, such as tumor stage and grade, are already known as prognostic factors for the survival of NETs [1]. Especially in pNET, genetic alterations have also been described as predictive for prognosis. Whole exome sequencing of pNET has recently demonstrated that about 43% of pNETs have somatic mutations in the genes *ATRX* (alpha-thalassemia/mental retardation syndrome X-linked) and/or *DAXX* (death domain associated protein) [12]. These recurrent loss-of-function mutations correlate with alternate lengthening of telomeres (ALT) and chromosomal instability [13,14]. The alterations of *ATRX*, *DAXX*, and *MEN1* are the most frequent mutations in sporadic pNET [12,15]. However, investigations of their prognostic value have yielded different results. Jiao et al. showed an association of *ATRX* or *DAXX* alterations with better survival [12]. Instead, Marinoni et al., Singhi et al., Yuan et al., and Chan et al. showed an unfavorable prognosis for pNETs characterized by *ATRX*, *DAXX*, or *MEN1* mutations [14,16,17,18]. However, it should be noted that Jiao et al. only used a small cohort of ten sequenced pNENs for their study [12]. In addition, the detection of ALT has been associated with a clinically more aggressive course with consequently a worse prognosis for patients affected by pNET [17]. The level of autophagic activity or the expression of autophagy-associated genes to influence survival in pNENs has not been investigated yet. It has only been observed that a lower expression level of autophagy-associated genes is associated with a metastatic stage [11].

The study aimed to determine the association between the expression of autophagy-associated gene transcripts, clinical parameters, and patient overall survival in pNENs tissue obtained from surgically resected specimens.

## 2. Results

A total of 54 specimens of pancreatic neuroendocrine neoplasms or their metastases were available for analysis from the tumor bank of the department of visceral, thoracic, and vascular surgery, which contained over 200 pNEN specimens at the time of analysis. The specimens were not selected according to a certain pattern. The included patients underwent surgical resection of their primary and/or metastases between 1989 and 2016. Patient characteristics are shown in Table 1. In total, 28/54 patients were female and 26/54 were male. The median age at diagnosis and the median age at surgery was 49.6 years. In total, 13/54 were non-functioning tumors.

In the study population, death was only observed in 12/54 patients (Table 1). The remaining patients were still alive at the time of analysis (*n* = 31) or got lost in the follow-up (*n* = 11). The median follow-up time was 8.6 years (range 0.003–21.912 years). The estimated median overall survival was not reached in the Kaplan–Meier analysis.

### 2.1. Autophagic Gene Expression in Sporadic and MEN1-Associated pNEN

**Figure 1:**
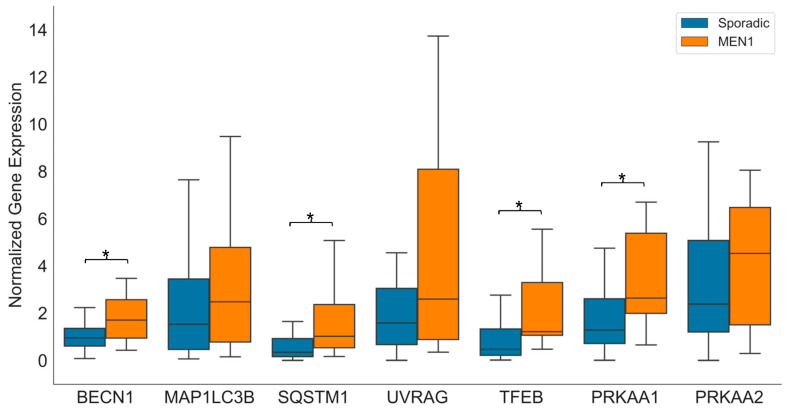
Box and whisker plot showing the expression of autophagic gene transcripts in sporadic pNEN (*n* = 40) and MEN1-associated pNEN (*n* = 14). * *p* < 0.05 was considered significant (two-sided Mann–Whitney U test). MEN1 = multiple endocrine neoplasia type 1.

The Mann–Whitney U test showed that pNEN patients with MEN1 syndrome (*n* = 14) tended to have a higher expression of all analyzed autophagic genes in comparison with patients affected by sporadic pNEN (*n* = 40). Significantly higher expression of *BECN1* (*p* = 0.046), *SQSTM1* (*p* = 0.01), *TFEB* (*p* = 0.005), and *PRKAA1* (*p* = 0.018) was observed in MEN1-associated pNEN (Figure 1).

The two-sided Mann–Whitney U test showed that a decreased expression of *MAP1LC3B* (*p* = 0.004), *SQSTM1* (*p* = 0.001), *TFEB* (*p* = 0.003), *PRKAA1* (*p* < 0.001), and *PRKAA2* (*p* = 0.015) were significantly associated with distant metastasis (*n* = 12) in pNEN. Instead, only a decreased expression of *SQSTM1* (*p* = 0.006), *TFEB* (*p* = 0.011), and *PRKAA1* (*p* = 0.001) could be observed in patients with lymph-node metastases (*n* = 17).

Non-functioning pNEN (*n* = 13) showed only a significantly lower expression of *TFEB* (*p* = 0.022) compared to functioning pNEN (*n* = 41).

According to univariable Cox regression, the expression level of autophagy-associated genes showed no significant prognostic value regarding overall survival after the initial diagnosis of pNEN (Table 2). Only the gender (male, *p* = 0.011), the evidence of lymph-node (*p* = 0.022) or distant metastasis (*p* = 0.001), the functional activity (*p* = 0.024), the tumor grade G2 (*p* = 0.018), the classification as pNEC (*p* = 0.001), or the evidence of infiltrative growth (*p* = 0.007) of the primary tumor were significant predictors of worse overall survival after the analysis performed by univariable Cox regression (Table 2). For univariable Cox regression, the high rate of loss to follow-up was considered a threat to validity.

Sporadic pNENs were further investigated separately regarding their expression of autophagic genes.

### 2.2. Autophagic Gene Expression in Sporadic pNEN

**Figure 2:**
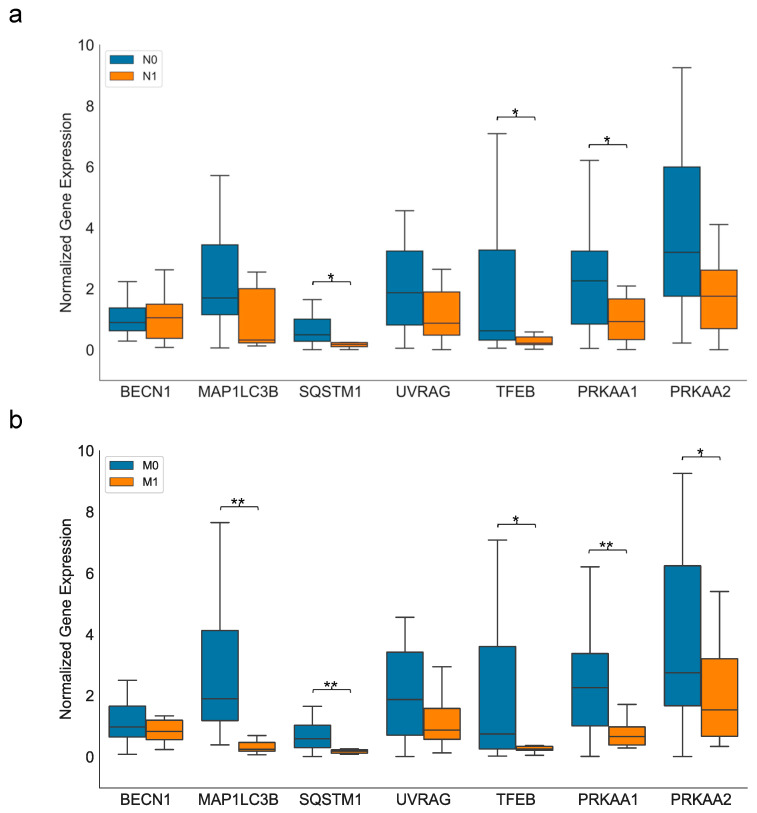
Box and whisker plot showing the expression of autophagic gene transcripts in sporadic pNEN. (**a**) N0 vs. N1; (**b**) M0 vs. M1; * *p* < 0.05 and ** *p* < 0.001 were considered significant in Mann–Whitney U test. N0 = no lymph node metastasis; N1 = lymph node metastasis; M0 = no distant metastasis; M1 = distant metastasis.

The clinical parameters of the patient diagnosed with sporadic pNEN (*n* = 40) were analyzed in correlation with the expression of autophagy-associated genes. The Mann–Whitney U test showed that the pNEN characterized by lymph node metastasis (N1, *n* = 14) showed a lower expression of most autophagy-associated genes compared to pNEN without lymph node metastasis (N0, *n* = 26), with *SQSTM1* (*p* = 0.01), *TFEB* (*p* = 0.023) and *PRKAA1* (*p* = 0.025) being significantly lower. Only *BECN1* expression was unchanged in both sample groups (Figure 2a).

The presence of distant metastasis (M1, *n* = 11) was also associated with a trend towards the lower expression of all autophagic gene transcripts, with *MAP1LC3B* (*p* < 0.001), *SQSTM1* (*p* < 0.001), *TFEB* (*p* = 0.013), *PRKAA1* (*p* < 0.001), and *PRKAA2* (*p* = 0.03) being significantly down-regulated (Figure 2b).

**Figure 3:**
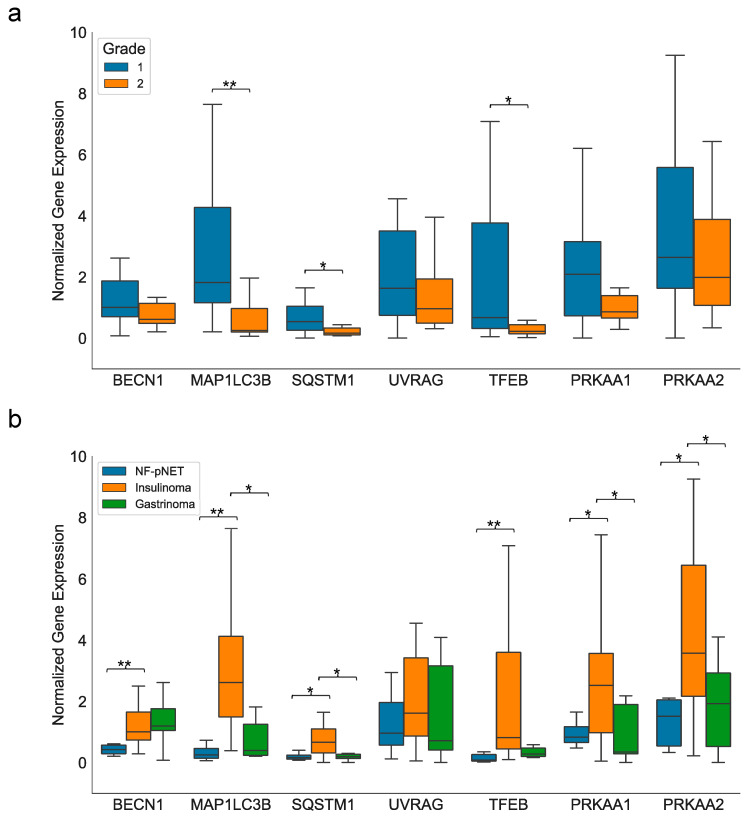
Box and whisker plots showing the expression of autophagic gene transcripts according to grading (**a**) or functionality (**b**) of sporadic pNETs; * *p* < 0.05 and ** *p* < 0.001 were considered significant in Mann–Whitney U test. NF-pNET = non-functioning pancreatic neuroendocrine tumor.

G1 pNETs (*n* = 27) were characterized by a significantly higher expression of *MAP1LC3B* (*p* < 0.001), *SQSTM1* (*p* = 0.025), and *TFEB* (*p* = 0.016) compared to G2 pNETs (*n* = 11) (Figure 3a). Differences to G3 pNETs and pNECs could not be determined due to the low sample size of pNECs (*n* = 2) and missing G3 pNETs in the study population.

Sporadic functionally active pNENs (*n* = 31) tended to show a higher expression level of the autophagic genes than NF-pNENs (*n* = 9). The expression levels of *BECN1* (*p* < 0.001), *MAP1LC3B* (*p* = 0.002), and *TFEB* (*p* = 0.001) were significantly higher in functionally active pNENs.

In particular, pancreatic insulinomas (*n* = 24) showed a higher expression of the autophagic genes *BECN1* (*p* < 0.001), *MAP1LC3B* (*p* < 0.001), *SQSTM1* (*p* = 0.008), *TFEB* (*p* < 0.001), *PRKAA1* (*p* = 0.038), and *PRKAA2* (*p* = 0.019) compared to NF-pNET (*n* = 7) (Figure 3b).

Interestingly, insulinomas (*n* = 24) evidenced a higher expression of autophagic genes than gastrinomas (*n* = 7). However, only *MAP1LC3B* (*p* = 0.022), *SQSTM1* (*p* = 0.014), *PRKAA1* (*p* = 0.014), and *PRKAA2* (*p* = 0.038) showed significantly higher expression (Figure 3b). No significantly different expression of autophagic genes could be identified between NF-pNET (*n* = 7) and gastrinomas (*n* = 7) (Figure 3b).

## 3. Discussion

Autophagy exerts a double-edged sword role in neoplastic diseases, because of its implication not only in tumor suppression but also in cancer survival [7]. Up to now, the significance of autophagy and the potential of its modulation have not yet been fully investigated in pancreatic neuroendocrine tumors. Autophagy inhibition through chloroquine induced apoptosis in well-differentiated pNEN cell lines via endoplasmic reticulum stress and resulted in decreased tumor size in *Men1*-heterozygous deficient mice, a mouse model for low-grade pNET [19]. A previous study of our group evidenced that prompting autophagy, by the administration of the pan-deacetylase inhibitor panobinostat, could lead to pNEN cell death, too [11]. The predictive value of autophagy markers and the significance of autophagy status for the treatment of pNEN are also still unknown. The proliferative rate and the tumor stage are currently most important factors for the prediction of the aggressive behavior of pNEN [20]. In the present study, we investigated whether the expression level of autophagic genes in human resected pNEN could represent a prognostic value for clinical aggressiveness.

This study showed that a higher expression level of autophagic genes is associated with less aggressive sporadic pNEN. G1 tumors were characterized by a higher expression of all autophagic genes compared to G2 sporadic pNENs. Furthermore, lymph node metastases and distant metastases occurred significantly more often in sporadic pNENs with a decreased level of autophagic genes.

The prognostic value of the expression of autophagy-associated transcripts has not been described to this extent in pNEN before. However, the key players of the PI3K-AKT-mTOR pathway, which negatively regulate autophagic activity, have already been identified as prognostic markers. Increased mTOR activity has often been observed in pNEN, most likely due to inactivating mutations of genes transcribing for negative regulators of the PI3K-AKT-mTOR pathway [15,21,22,23].

The mutation-related functional impairment of the mTOR inhibitors, phosphatase and ten-sin homolog (PTEN), or tuberous sclerosis complex 2 (TSC2) are already known as characteristic genetic alterations occurring in pNEN [12,15,16,23]. The low expression of these endogenous mTOR inhibitors was associated with more aggressive pNEN, decreased disease-free survival, and overall survival [23,24]. The increased activity of the PI3K-AKT-mTOR pathway was shown to be prognostically unfavorable [23,24].

Even within the low-grade (G1) pNENs group, the loss of *PTEN* and the consecutive upregulation of pAKT were found to be associated with a more aggressive clinical course [25]. Instead, the pancreatic islets cells showed strong immunohistochemical staining for PTEN and mTOR with an absent expression of the activated mTOR [24].

Notably, within the sporadic pNENs, insulinomas expressed high levels of autophagic transcripts that distinguished them from gastrinomas and non-functional pNETs. However, a different frequency of mutations with a consecutive increase in PI3K-AKT-mTOR activity has not been evidenced between insulinomas, gastrinomas, and NF-pNETs yet.

In addition, the pNENs associated with MEN1-syndrome of our study population significantly differ from sporadic pNENs concerning the expression of autophagic genes. Interestingly, it was observed that MEN1-associated pNENs tend to show a higher expression of autophagic genes than sporadic pNENs.

Up to 44% of sporadic pNENs are known to carry somatic inactivating mutations in the *MEN1* gene, too [12,26], but MEN1 syndrome-associated pNENs do not frequently show alterations of the autophagy-associated genes or the PI3K-AKT-mTOR signaling pathway. The frequency of *PTEN* and *TSC2* mutations in sporadic pNENs may cause the lower expression level of autophagic genes found in this study via the increased autophagy inhibitory activity of the PI3K-AKT-mTOR pathway. However, the results of this preliminary study are limited, and further analyses are necessary to confirm such a hypothesis.

The association of autophagic gene expression with overall survival can only be assessed to a limited extent due to the small number of events. The median overall survival wasn’t reached. But most importantly, many patients received their follow-ups outside of the study center. This resulted in a relatively large number of patients being lost to follow-up and a serious threat to the validity of survival analysis [27]. Therefore, autophagic gene expression must be analyzed in a larger study population with less loss to follow-up. Furthermore, the evaluation of the prognostic relevance of autophagic genes for the clinical aggressiveness of pNEN is limited due to missing G3 pNET and the low number of pNEC in the study population. Sample collection was predominantly in the context of curative surgery, resulting in a selection bias for the study population.

Due to the mostly curative surgical therapy, the study population does not allow a retrospective comparison of the response to systemic therapies with the expression level of the autophagic genes. A small number of the samples was obtained from metastases instead of primaries. This limits the homogeneity of the study group due to possible mutational variation between primaries and metastases.

Furthermore, it has not been investigated whether the expression level of autophagic genes correlates with an induction of autophagy in pNENs. Only a previous in vitro study with pNEN spheroids has shown, so far, that the increased expression of autophagic gene transcripts was associated with an increase in the autophagic process [11].

Despite these limitations, this preliminary study highlights a significant difference in autophagy gene expression patterns between G1 and G2 sporadic pNET and between non-metastatic and metastatic sporadic pNEN. The assessment of the autophagy status could be an interesting approach to subtyping neuroendocrine neoplasms. However, the prognostic value and possible therapeutic consequences of the autophagy status in pNEN need to be further tested.

The different expression of autophagic genes within NENs, in addition to their prognostic value, could be a promising approach for precision medicine related to pNEN.

The autophagic status has not been considered in analyses related to the treatment benefits of therapies in human NEN before. However, Gelsomino et al. showed in a retrospective analysis of a small cohort of 24 patients (NEN) treated with the mTOR inhibitor everolimus that an immunohistochemically positive expression of the autophagy negative regulator pmTOR/p-S6K is associated with better OS than in pmTOR/p-S6K negative patients.

In the Rip1Tag2 mouse model, the induction of autophagy was found to be a resistance mechanism to sunitinib [9]. Whether certain patients with metastatic pNEN benefit from additional treatment with an autophagy inhibitor in their systemic therapy has not been investigated yet.

Even though tumor grade and stage are established markers for therapy decision in pNEN, the step toward personalized therapy is challenging due to the heterogeneity of pNEN and the lack of molecular markers. The autophagy status may be a future target and could improve treatment decisions.

## 4. Materials and Methods

### 4.1. Study Population, Processing, and Analysis of Human pNEN Specimens

The tumor bank of the department of visceral, thoracic, and vascular surgery was searched for available human pNEN specimens of patients who underwent surgical resection of primary and/or metastases. All patients who underwent surgery after 1999, gave written consent for the use of the resected tissue for scientific purposes. According to the Ethics Committee of the Philipps University of Marburg, no written consent for the scientific use of resected tissues, which were obtained before the year 2005, is needed. The processing of the tissues and the analysis of the medical data is carried out after approval by the Ethics Committee of the Philipps University of Marburg (104/99). RNA was isolated from these snap-frozen pNEN specimens according to the manufacturer’s protocol of the RNA RNeasy Mini Kit (74106; Qiagen, Hilden, Germany). Reverse transcription of the isolated mRNA was performed using the iScript cDNA Synthesis Kit (170–8891; Bio-Rad, Dreieich, Germany) in a FlexCycler (Analytik Jena AG, Jena, Germany). RT-qPCR was performed by using the GoTaq qPCR Master Mix (Promega, Madison, WI, USA) in a RT-qPCR Thermocycler CFX96 Real-Time System (Bio-Rad Laboratories), and with the following Qiagen human primers: *BECN1* (QT00004221), *MAP1LC3B* (QT00034328), *SQSTM1* (QT00095676), *UVRAG* (QT00034328), *TFEB* (QT00069951), *PRKAA1* (QT00009436), *PRKAA2* (QT00042077), and *GAPDH* (QT01192646).

RT-qPCR results were analyzed by using Bio-Rad CFX Manager software (Bio-Rad Laboratories, Hercules USA) and normalized to the *GAPDH* expression of each sample. Further, commercially available (Lonza, Basel, Switzerland) physiological pancreatic islets of Langerhans (2000) from a healthy donor were used as a control. RNA was isolated and RT-qPCR was performed according to the methods described above. The expression levels of the autophagy-associated gene transcripts of the human pNEN specimens were then normalized against the expression level of the control. The following criteria were fulfilled by the included patients: (1) histologically confirmed pancreatic or duodenal neuroendocrine tumor; (2) no second malignancy.

Patient characteristics were respectively obtained from the medical record. More aggressive pNEN are usually characterized by poorer outcomes compared to pNEN with indolent behavior. Due to limited survival data of the study population, metastatic disease and higher grading were considered characteristics of pNEN with more aggressive behavior [28,29,30].

### 4.2. Data Analysis

Missing data were analyzed before evaluating the data. The missing data analysis for continuous variables revealed missing data for OS at the time of diagnosis (7.4%), OS at the time of surgery (7.4%), age at surgery (5.6%), age at diagnosis (5.6%), *BECN1* (1.9%), *UVRAG* (7.4%), *TFEB* (1.9%), *PRKAA1* (1.9%), and *PRKAA2* (5.6%). These missing values were determined to be missing completely at random by Little’s missing completely at random (MCAR) test and were handled by using multiple imputation before further analysis [31]. The only categorial variable with missing values was the grading of seven highly differentiated pNETs. The missing values were filled, arbitrarily, with the most frequent value (G1). After handling the missing data, further analyses were conducted to determine the value of autophagy-related gene expression in pNEN.

The expression of autophagy-associated transcripts showed no normal distribution by using visual (histograms, probability-probability(Q-Q) plots) and analytical methods (Kolmogorov–Smirnov/Shapiro–Wilk Test). Therefore, the Mann–Whitney U test was performed as a two-sided test to assess significant differences between subgroups regarding the expression of autophagy-associated transcripts.

The Kaplan–Meier method was used to estimate the median overall survival. A univariable Cox regression analysis was conducted to determine the prognostic value of clinical parameters and the expression level of the autophagy-associated transcripts regarding OS. Statistical analyses were conducted using IBM SPSS Statistics 27 (IBM, Armonk, NY, USA), and *p* < 0.05 was considered significant.

## 5. Conclusions

This study shows that the reduced expression of autophagic genes is associated with the metastatic stage of sporadic pNEN.

## Figures and Tables

**Table 1:** Patients’ characteristics.

	Patients’ Characteristics, *n* (%)
Sex	
male	26 (48.1%)
female	28 (51.9%)
Age at diagnosis	
<50	29 (53.7%)
≥50	25 (46.3%)
Age at surgery	
<50	26(%)
>50	28(%)
Specimen	
Primary	51 (94.4%)
Lymph node metastasis	2 (3.7%)
Liver metastasis	1 (1.9%)
Grading	
G1	38 (70.4%)
G2	14 (25.9%)
G3	2 (3.7%)
Infiltrative growth	
Yes	3 (5.6%)
No	51 (94.4%)
Hormonal activity	
non-functional pNEN	13 (24.1%)
NF-pNET	11 (20.4%)
NEC	2 (3.7%)
functional pNEN	41 (75.9%)
insulinoma	29 (53.7)
gastrinoma	12 (22.2%)
Metastatic sites	
N0	37 (68.5%)
N1	17 (31.5%)
M0	42 (74.1%)
M1	12 (22.2%)
Sporadic pNEN	40 (74.1%)
MEN1 associated pNEN	14 (25.9%)

pNEN: pancreatic neuroendocrine neoplasm; pNET: pancreatic neuroendocrine tumor; NF-pNET: non-functional pancreatic neuroendocrine tumor, G = grade; N0 = no lymph node metastasis; N1 = lymph node metastasis; M0 = no distant metastasis; M1 = distant metastasis.

**Table 2:** Prognostic value of patient’s characteristics and autophagic transcript expression regarding overall survival in pNEN (*n* = 54). * *p* < 0.05. Due to the high rate of loss to follow-up (18%), the risk of poor validity must be considered.

	Univariable Cox Analysis	
Variable	Hazard Ratio (95% CI)	*p*-Value
Female (versus male)	0.14 (0.031–0.641)	0.011 *
Age at diagnosis	1.026 (0.983–1.07)	0.238
Age at surgery	1.028 (0.984–1.073)	0.217
Grading (versus G1)		<0.001 *
G2	4.576 (1.292–16.205)	0.049 *
G3	44.302 (6.215–315.771)	<0.001 *
NEC (versus pNET)	23.224 (3.803–141.835)	0.001 *
Functional pNET (*versus* non-functional pNET)	0.268 (0.085–0.838)	0.024 *
Ref. NF-pNET		0.004 *
Insulinoma	0.26 (0.058–1.168)	0.079
Gastrinoma	0.646 (0.143–2.915)	0.57
pNEC	11.966 (1.72–83.237)	0.012 *
Lymph node metastasis	3.836 (1.209–12.169)	0.022 *
Extrahepatic metastasis	8.097 (2.474–26.499)	0.001 *
MEN1 syndrome associated pNEN (*versus* Sporadic pNEN)	0.213 (0.028–1.655)	0.139
Infiltrative growth	8.567 (1.79–40.995)	0.007 *
Expression of autophagy associated transcripts		
BECLIN 1	0.489 (0.201–1.189)	0.114
MAP1LC3B	0.801 (0.573–1.12)	0.194
SQSTM1	0.373 (0.1–1.393)	0.142
UVRAG	0.953 (0.836–1.087)	0.475
TFEB	0.396 (0.135–1.163)	0.092
PRKAA1	0.75 (0.513–1.097)	0.138
PRKAA2	1.002 (0.888–1.131)	0.968

Ref. = reference; G = grade; pNET = pancreatic neuroendocrine tumor; pNEN = pancreatic neuroendocrine neoplasm; pNEC = pancreatic neuroendocrine carcinoma; MEN1 = multiple endocrine neoplasia type 1.

## Data Availability

The RT-qPCR raw data presented in this study are available on request from the corresponding author.
